# Sentinel lymph node mapping in endometrial cancer: a systematic review and meta-analysis

**DOI:** 10.18632/oncotarget.16662

**Published:** 2017-03-29

**Authors:** Hefeng Lin, Zheyuan Ding, Vishnu Goutham Kota, Xiaoming Zhang, Jianwei Zhou

**Affiliations:** ^1^ School of Medicine, Zhejiang University, Hangzhou 310058, China; ^2^ Zhejiang Provincial Center for Disease Control and Prevention, Hangzhou 310051, China; ^3^ Department of Gynecology, The Second Affiliated Hospital, School of Medicine, Zhejiang University, Hangzhou 310009, China

**Keywords:** endometrial cancer, sentinel lymph node mapping, detection rate, sensitivity, meta-analysis

## Abstract

Endometrial cancer is the most frequent tumor in the female reproductive system, while the sentinel lymph node (SLN) mapping for diagnostic efficacy of endometrial cancer is still controversial. This meta-analysis was conducted to evaluate the diagnostic value of SLN in the assessment of lymph nodal involvement in endometrial cancer. Forty-four studies including 2,236 cases were identified. The pooled overall detection rate was 83% (95% CI: 80–86%). The pooled sensitivity was 91% (95% CI: 87–95%). The bilateral pelvic node detection rate was 56% (95% CI: 48–64%). Use of indocyanine green (ICG) increased the overall detection rate to 93% (95% CI: 89–96%) and robotic-assisted surgery also increased the overall detection rate to 86% (95% CI: 79–93%). In summary, our meta-analysis provides strong evidence that sentinel node mapping is an accurate and feasible method that performs well diagnostically for the assessment of lymph nodal involvement in endometrial cancer. Cervical injection, robot-assisted surgery, as well as using ICG, optimized the sensitivity and detection rate of the technique. Sentinel lymph mapping may potentially leading to a greater utilization by gynecologic surgeons in the future.

## INTRODUCTION

Endometrial cancer is the most frequently tumor of the female reproductive system in developed countries with an approximately 60,050 cases and 10,470 deaths for the year 2016 in the United States [1]. The surgical management of endometrial cancer is still controversial, especially in the early stage. The study shows that a complete lymphadenectomy may have no therapeutic benefit in patients with early-stage endometrial cancer [2]. Comprehensive lymphadenectomy not only increases operative time and blood loss but also is associated with surgical complications, such as blood vessel and nerve damage, lymphoedema, and lymphocyst formation [3]. The rate of long-term lymphedema directly attributed to lymphadenectomy was recently reported to be 23% [4]. Furthermore, lymphadenectomy imposes significant morbidity for the patients [3].

Lymph node status is a major prognostic factor and a criterion for adjuvant therapy in endometrial cancer. The concept of the sentinel lymph node (SLN), the node(s) most likely to harbor the first metastasis from the primary tumor, was first introduced in 1960 following observations associated with parotid gland carcinomas [5]. As the sentinel lymph node is relatively the first in a chain of lymph nodes, theoretically the sentinel node will be the first to encounter the effects of the metastatic form of the disease. If the sentinel node is negative, then it can be safely assumed that the remainder of the lymphatic basin is also unaffected by metastasis. The advantage of the distinctive benefits of SLN mapping is the opportunity to avoid “over-staging”, leading to a relatively lower morbidity than in the case of the performance of a full lymphadenectomy and the potential for improved diagnostic accuracy [6]. As a surgical technique, the SLN mapping has been implemented in the standard of treatment for patients with melanoma and breast cancer [7].

If the SLN concept is valid in endometrial cancer, most patients, especially women affected with endometrial cancer in the early stage could avoid the risks associated with the lymphadenectomy. However, the diagnostic efficacy of SLN mapping in endometrial cancer is still controversial. Therefore, we performed a meta-analysis to evaluate different sentinel lymph node mapping techniques and their corresponding detection rates and sensitivity in endometrial cancer.

## RESULTS

**Figure 1:**
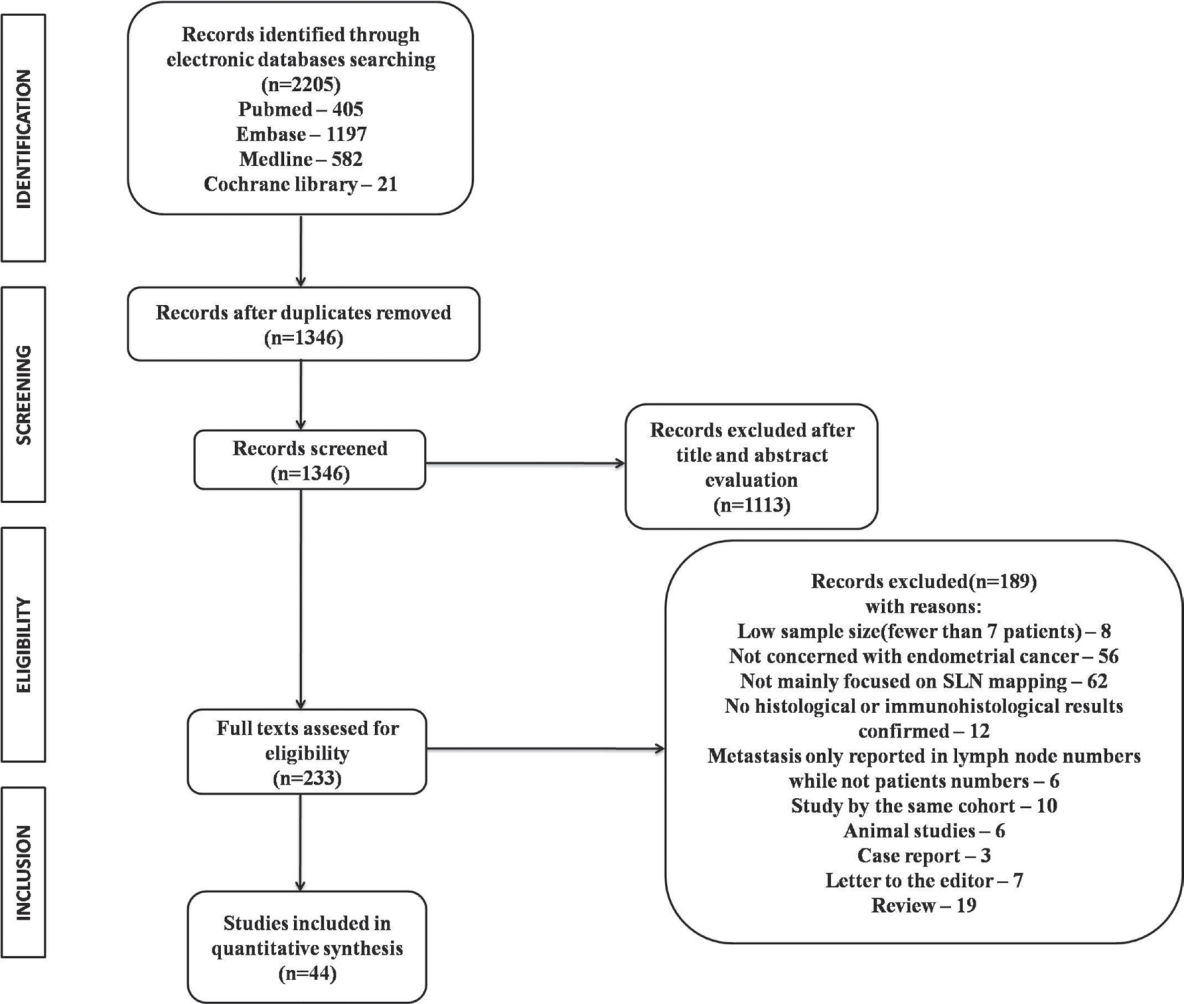
Flow diagram of studies identified, included, and excluded

Overall, 2,205 studies were retrieved through the electronic databases searching. Among these, 859 (39.0%) studies were removed as duplicates. After title and abstract evaluation, 233 (10.6%) remained for full review. Of these, 189 (8.6%) full-text articles were additionally excluded for the following reasons: 8 (0.36%) had low sample size(fewer than 7 patients); 56 (2.5%) not concerned with endometrial cancer; 62 (2.8%) not mainly focused on SLN mapping; 12 (0.54%) without histological or immunohistological results; 6 (0.27%) reported only the numbers of metastasized lymph nodes but not the number of patients; 6 (0.27%) from the same research team using the same database; 10 (0.45%) were animal studies; 3 (0.1%) were case reports; 7 (0.32%) were letters; 19 (0.86%) were reviews. Thus, 44 (2.0%) studies [11–54] were deemed as eligible by the authors (involving 2,236 patients) after conducting a comprehensive and through literature search. Figure 1 shows the process involved in the assessment of the studies and in accordance to the process highlighted in the figure the studies were identified, those of them that fulfilled the conditions detailed earlier were included and those that didn't were excluded.

### Sensitivity

**Figure 2:**
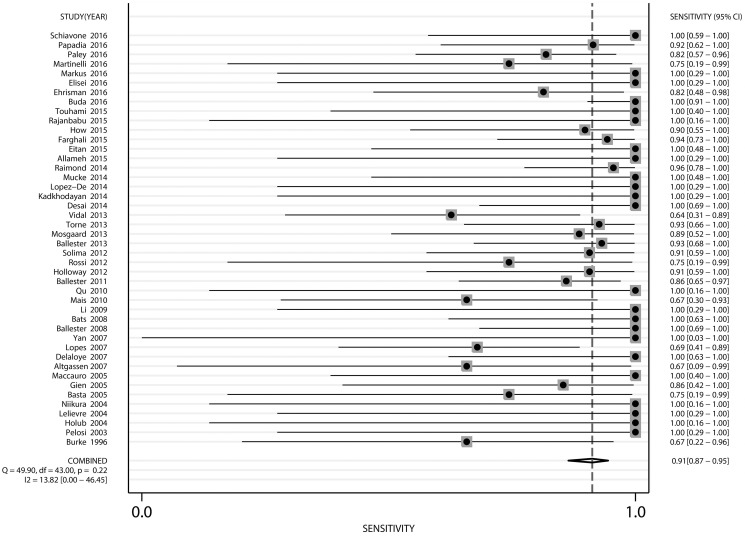
Forest plot of pooled sensitivity and 95% CI in SLN mapping in endometrial cancer

The pooled sensitivity for all studies deemed eligible (*n* = 44) by the authors was 91% (95% CI: 87–95%), the Cochran Q value was 49.90 *(p* = 0.22 and *I*^2^ = 13.82%). The forest plot of sensitivity pooling is shown in Figure 2.

**Figure 3:**
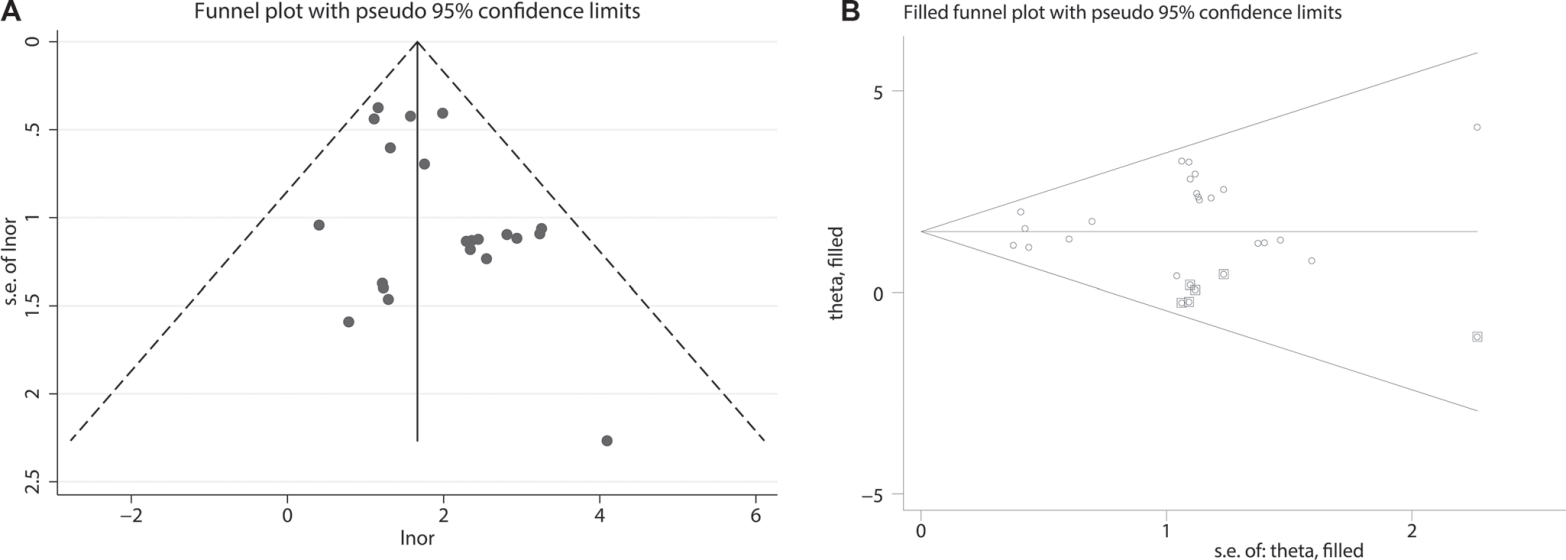
(**A**) Funnel plot of pooled sensitivity. (**B**) Funnel plot of pooled sensitivity by using trim and fill method.

The funnel plot of the sensitivity pooling and the funnel plot of the sensitivity pooling by using trim and fill method are shown in Figure 3. The egger's regression intercept was found out to be 2.34 (*p* = 0.031).

### Overall SLN detection rate

**Figure 4:**
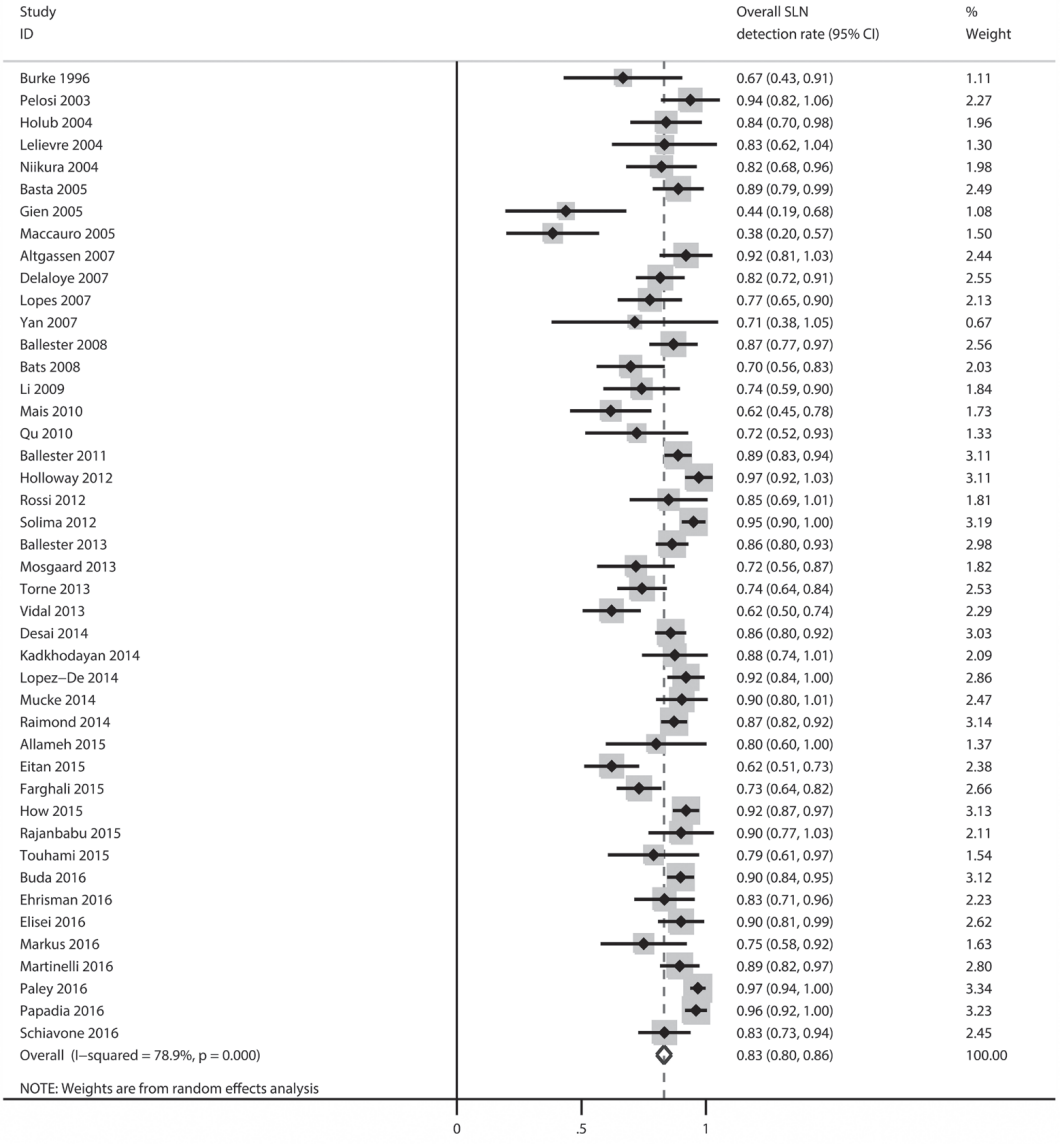
Forest plot of pooled overall detection rate and 95% CI in SLN mapping in endometrial cancer

Figure 4 shows the forest plot associated with the overall SLN detection rate. The pooled detection rate was 83 % (95% CI: 80–86%), with heterogeneity *I*^2^ = 78.9% (*p* = 0.000).

**Figure 5:**
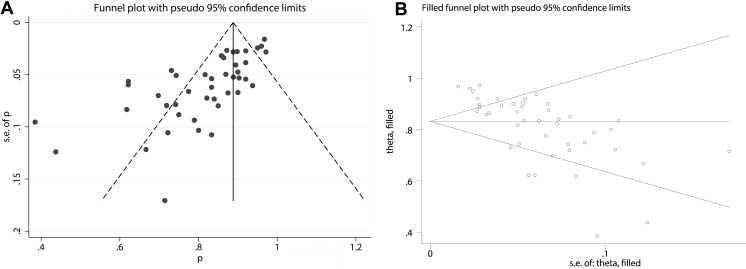
(**A**) Funnel plot of pooled overall SLN detection rate. (**B**) Funnel plot of pooled overall SLN detection rate by using trim and fill method.

The funnel plot of the overall SLN detection rate pooling and the funnel plot of the overall SLN detection rate pooling are shown in Figure 5. The egger's regression intercept was −7.06 (*p* = 0.000).

### Bilateral SLN detection rate

**Figure 6:**
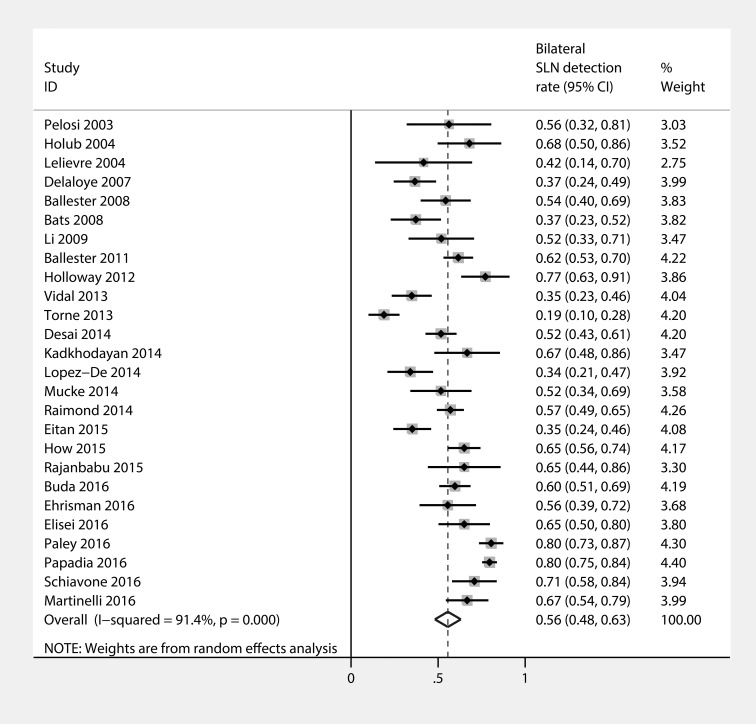
Forest plot of pooled bilateral SLN detection rate and 95%CI in SLN mapping in endometrial cancer

Figure 6 shows the forest plot of the bilateral SLN detection rate. The bilateral SLN detection rate was observed to be 56% (95% CI: 48–63%), with heterogeneity *I*^2^ = 91.4% (*p* = 0.000).

### Sub-group analysis

The sentinel lymph node detection rate and sensitivity were related to the mapping method employed (blue dye alone, radiotracer dye with blue dye, ICG), the surgical approach followed (laparotomy, laparoscopy, robotic assistance) and the site of dye injection (cervical injection, uterine injection) (Table 1). Use of ICG showed high pooled overall detection rate: 93% (95% CI: 89–96%) and high pooled bilateral detection rate: 78% (95% CI: 72–84%). The Robot-assisted surgery also had high pooled overall detection rate: 86% (95% CI: 79–93%) and pooled bilateral detection rate: 62% (95%CI: 43–80%). The sensitivity and overall detection rate were high in patients in whom cervical injection was the site of dye injection 93% (95% CI: 87–96%) and 86% (95% CI: 83–89%), respectively and both blue dye and radiotracer dye were used for mapping 92% (95% CI: 84–96%) and 86% (95% CI: 82–90%), respectively. Laparoscopic surgery showed higher pooled sensitivities 96 % (95% CI: 88–99%).

**Table 1:** Results of sub-group analysis

Sub-group		Detection rate (95% CI)	Sensitivity (95% CI)	Bilateral detection rate (95% CI)
Surgical approach	Laparoscopy	82% (78–87%)	96% (88–99%)	58% (47–69%)
	Laparotomy	77% (71–84%)	89% (80–95%)	47% (32–61%)
	Robotic assistance	86% (79–93%)	90% (77–96%)	62% (43–80%)
Mapping method	Blue dye	76% (71–81%)	90% (79–96%)	44% (38–50%)
	ICG	93% (89–96%)	87% (76–93%)	78% (72–84%)
	Tc-99m+blue dye	86% (82–90%)	92% (84–96%)	56% (41–71%)
Injection site	Cervical injection	86% (83–89%)	93% (87–96%)	60% (52–69%)
	Uterine injection	76% (68–83%)	88% (78–93%)	47% (11–84%)

Abbreviations: ICG, indocyanine green; Tc-99m, technecium-99.

## DISCUSSION

To our knowledge, this is the newest meta-analysis focused on the diagnostic efficacy of sentinel lymph node mapping in endometrial cancer. In our meta-analysis of 44 studies comprising 2,236 cases, sentinel node mapping yielded a pooled detection rate of 83%. The pooled sensitivity implies that 91% of occult lymph node metastases could be diagnosed by SLN mapping, which seems to be equivalent to those achieved in patients with breast cancer (sensitivity 91%) [55]. However, bilateral nodes were detected in only 59% (26 studies) of the patients. The Endometrium as a midline organ and it exhibits two different pathways of lymphatic drainage: right and left [28]. The bilateral detection method has been used for other midline organs such as the penis with fairly promising results [56]. In the present study,the rate of bilateral SLN detection was between 89% (in the study conducted by Martinelli et al.) [51] and 19% (in the study conducted by Torné et al.) [34], and the pooled bilateral detection rate was 56% (95% CI: 48–64%). Therefore, achieving high bilateral SLN detection rates of endometrial cancer is mandatory to implement the SLN mapping as a routine component of clinical practice.

It should be mentioned that in the previous meta-analyses focusing on the diagnostic efficacy of SLN mapping in endometrial cancer, Kang et al. [57] reported a pooled detection rate of 78% (95% CI: 73–84%) (*n* = 1,101), while Ansari et al. [58] reported a pooled detection rate and sensitivity of 77.8% (95% CI: 74–82%) and 89% (95% CI: 83–93%) respectively (*n* = 2,071). Compared to these previous meta-analyses, our research found higher detection rate of 83% (95% CI: 80–86%) (*n* = 2,236) and sensitivity of 91% (95% CI: 87–95%). Since previous two meta-analyses were published several years ago, our meta-analysis included the newest results of the recent studies, which makes it more valid.

In addition, the studies included in our systematic review were heterogeneous and therefore gave rise to the need for conducting a sub-group analysis to explore the reasons for the observed heterogeneity. Laparoscopic surgery and robot-assisted surgery, were associated with high detection rates and sensitivities when compared with an open surgery based approach. The pilot study conducted by Mais et al. [26] showed that the high detection rates obtainable through laparoscopy were not reproducible through laparotomy. The different detection rates observed through laparoscopy or through laparotomy might depend on the different time intervals elapsing between the injection of the vital dye into the cervix and the surgical SLN assessment in the pelvic basin. In fact, this time interval was always found to be shorter in the case of laparoscopy than for laparotomy. The current study also showed that the pooled sensitivity and detection rates in endometrial cancer patients are high for SLN mapping when the mapping was carried out using ICG. When the mapping was carried out using solely the blue dye, the pooled detection rate was observed to be rather low. Moreover, there are some disadvantages of the use of the blue dye. Allergic reactions to the blue dye were observed in 0.14–3% of the patients, including urticaria, skin rashes, erythema, blue hives, cardiovascular collapse, and anaphylactic shock. Other side effects include temporary skin tattooing, blue discoloration of the operative field following peritumoral injection, blue-colored urine for up to 24 hrs. following administration, and a factitious drop in intraoperative oxygen saturation measured by pulse oximetry. Furthermore, the teratogenicity and the long-term toxicity associated with the blue dye are unknown and could have serious effects on pregnancy [59, 60]. Indocyanine green (ICG) represents a feasible alternative to the more traditional methods of sentinel lymph node (SLN) mapping, and the interest associated with this promising tracer is growing. A recent meta-analysis conducted by Ruscito et al. [61] demonstrated that ICG SLN mapping seems to be equivalent to the combination of blue dyes and Tc-99m in terms of overall and bilateral detection rates in uterine malignancies. The good toxicity profile and ease of use of ICG, which does not require the injection to be administered in a controlled environment is also very desirable. Another issue in SLN mapping pertains to the identification of the optimal injection site for the radiocolloid /dye in patients with endometrial cancer. A comprehensive sub-group analysis showed that the use of cervical injection as the dye injection approach was found to cause an increase of 10% and 5% in the detection rate and sensitivity respectively, as compared to the use of uterine injection as the dye injection approach. It seems to be a lack of sufficient consensus about the best site of injection. The cervical injection approach is a method which is easy to carry out, reproducible, and is very suitable for standardization.

The meta-analysis performed during the course of this study has the following limitations that must be taken into account. First, the results presented in the current study were based on unadjusted estimates; more accurate outcomes would result from the adjustments made to be considered for other confounders such as age, body mass index, cancer stage, and so on. Second, the studies included in this analysis were not sufficient enough, especially from the perspective of a subgroup analysis. Thus, a potential publication bias is very likely to be associated with the results provided in this study in spite of evidence obtained from the statistical tests performed. Finally, only English and Chinese reports have been included in the analysis and consequently this might lead to the study not considering the data from other relevant studies published in other languages, which may result in causing a potential language bias.

In conclusion, the present results confirmed that sentinel lymph node mapping is a feasible and reliable approach that performs well diagnostically for the assessment of lymph nodal involvement in endometrial cancer. We also found that SLN mapping using some new techniques, such as ICG and robot-assisted surgery demonstrated higher detection rates compared to other modalities. The use of cervical injection and the mode of dye injection for both the blue dye and the radiotracer of the mapping material can optimize the sensitivity and detection rate of the technique. Further clinical trials are required to investigate the relationship between lymphadenectomy guided by SLN mapping and prognosis of endometrial cancer in the future.

## MATERIALS AND METHODS

### Search strategy

A comprehensive literature search on the retrieved publications (the last search was done in November, 2016) was performed independently by two authors associated with this current study. The language of studies was limited to English and Chinese only. The primary sources for the literature search were the electronic databases: Pubmed, Embase, Medline and the Cochrane Library. The predefined keywords used for the search were “sentinel lymph node” and “endometrial cancer”. A search algorithm that selected and screened results based on a combination with the following search terms: “sentinel AND (endometri* OR uterus OR uterine OR corpus uteri ) AND (cancer OR neoplasm* OR carcinoma* OR malignanc* OR tumo*)” was used to perform the literature search detailed in this study.

### Inclusion and exclusion criteria

For evaluating the diagnostic performance of sentinel lymph node mapping in endometrial cancers, the studies in accordance to the following inclusion criteria were included: (1) Studies with the enrollment of at least 7 women diagnosed with endometrial cancer; (2) The SLN mapping was the study's primary focus; (3) Studies validated by pelvic with/without para-aortic lymph node dissection and pathological examination including H&E (hematoxylin-eosin) staining or immunohistochemistry (IHC) were taken as the reference standard; (4) SLN mapping as the diagnostic method; (5) Studies where the total number of enrolled patients as well as those with detected SLN were both reported; (6) Studies that reported the total number of patients with a positive lymph node diagnosis, as well as those with false negative results.

Review articles, letters, comments, conference proceedings, unpublished data and case-reports were excluded. To avoid overlapping patient data in duplicate publications, we included the more recent articles with the largest sample sizes.

### Study quality assessment

The quality assessment of studies included in this article was undertaken by authors Lin and Zhang. The “QUADAS-2” (Quality Assessment of Diagnostic Accuracy Studies-2) tool, an official tool for assessing the quality of the diagnosis accuracy of a study (launched in 2011) was used to assist with the above-mentioned quality assessment [8]. The core “QUADAS-2” items used in our study are detailed outlined in the Appendix Table 1. “QUADAS-2” divided the assessment items into the risk of bias and the applicability, and has several items including: patient selection, index test, reference standard, flow and timing. The criteria could be scored as “yes”,”no”, or “not reported” in the publication.

### Data abstraction

Data from the included studies were extracted and summarized independently by the two authors mentioned earlier (Lin and Zhang). The extracted data primarily included (Supplementary Table 1): first author, publication year, country, sample size of the study, detection rate, tracer, injection site, pathology assessment and surgical approach. Wherever possible, the SLN detection rate was calculated in patients with bilateral sentinel node identification. The sensitivity associated with the sentinel lymph node procedure was defined as the total number of true positives in patients with a positive histopathology. A positive sentinel node was considered as true positive (TP) irrespective to the status of the other nodes, and a true negative (TN) was a negative sentinel node only if all other non-sentinel nodes were negative. A false negative (FN) was defined as positive non-SLN with negative SLN. The SLN detection rate can be defined as the percentage of patients in which at least one SLN was identified.

### Statistical analysis

The statistical analysis tool, Stata 12.0 software (StataCorp, College Station, Texas, USA) was employed to perform the aggregate data meta-analyses and evaluate the heterogeneity of the included studies. The random effects model was applied for calculating the overall detection rate, the bilateral detection rate, and the sensitivity from the data provided in the source articles. The results were depicted graphically as forest plots. The pooled data was presented with 95% confidence intervals (95% CI). The potential heterogeneity of the sensitivity and the detection rate was analyzed with the Cochrane *Q* test (the *p*-values less than 0.05 were considered as statistically significant). *I*^2^ index was used to quantify the heterogeneity, analyzing how much of the variance associated with the included studies was real and wasn't due to sampling errors. Funnel plots, Egger's regression intercepts [9], and the Duval and Tweedie's [10] “trim and fill” method were used for the evaluation of publication bias. A subgroup analysis was performed for expolring the heterogeneity in three variables: surgical approach, mapping method, injection site.

## SUPPLEMENTARY TABLE AND APPENDIX TABLE






