# Supplementary material for: Sentinel lymph node mapping in endometrial cancer: a systematic review and meta-analysis
Source: Oncotarget. 2017 Mar 29;8(28):46601–10. doi: 10.18632/oncotarget.16662 (PMC5542296; doi:10.18632/oncotarget.16662)
Supplement: Supplementary file 1 [file oncotarget-08-46601-s001.pdf]

Sentinel lymph node mapping in endometrial cancer: a systematic review and meta-analysis

Supplementary Materials

Supplementary Table 1: Characteristics of included studies in the meta-analysis. See Supplementary_Table_1

Appendix Table 1: Quality assessment of included studies (The QUADAS-2 tool). See Appendix_Table_1
