# Supplementary material for: Sentinel lymph node mapping in endometrial cancer: a systematic review and meta-analysis
Source: Oncotarget. 2017 Mar 29;8(28):46601–10. doi: 10.18632/oncotarget.16662 (PMC5542296; doi:10.18632/oncotarget.16662)
Supplement: Supplementary file 2 [file oncotarget-08-46601-s002.docx]

**Table 1: Characteristics of included studies in the meta-analysis**

| **First author** | **Publication**  **year** | **Country** | **Study size** | **Overall SLN DR** | **Bilateral SLN DR** | **Tracer** | **Injection site** | **Pathology assessment** | **Type of surgery** |
| --- | --- | --- | --- | --- | --- | --- | --- | --- | --- |
| Burke [11] | 1996 | United States | 15 | 0.67 | NR | Blue dye | Uterine | H&E | Open |
| Pelosi [12] | 2003 | Italy | 16 | 0.94 | 0.56 | Tc-99m+blue dye | Cervical | H&E,IHC | Laparoscopic |
| Holub [13] | 2004 | Czech Republic | 25 | 0.84 | 0.68 | Tc-99m+blue dye/ICG | Cervical | H&E | Laparoscopic |
| Lelievre [14] | 2004 | France | 12 | 0.83 | 0.42 | Tc-99m+blue dye | Cervical | H&E,IHC | Laparoscopic |
| Niikura [15] | 2004 | Japan | 28 | 0.82 | NR | Tc-99m+blue dye | Cervical/Uterine | H&E,IHC | Open |
| Basta [16] | 2005 | Poland | 36 | 0.89 | NR | Tc-99m+blue dye/ICG | Cervical | H&E,IHC | NR |
| Gien [17] | 2005 | Canada | 16 | 0.44 | NR | Blue dye | Uterine | H&E | Open |
| Maccauro [18] | 2005 | Italy | 26 | 0.38 | NR | Tc-99m+blue dye | Uterine | H&E,IHC | Laparoscopic |
| Altgassen [19] | 2007 | Germany | 25 | 0.92 | NR | Blue dye | Uterine | H&E | Open |
| Delaloye [20] | 2007 | Switzerland | 60 | 0.82 | 0.36 | Blue dye | Uterine | H&E,IHC | Open |
| Lopes [21] | 2007 | Brazil | 40 | 0.78 | NR | Blue dye | Uterine | H&E,IHC | Open |
| Yan [22] | 2007 | China | 7 | 0.71 | NR | Blue dye | Cervical | H&E | Laparoscopic |
| Ballester [23] | 2008 | France | 46 | 0.87 | 0.63 | Blue dye | Cervical | H&E,IHC | Laparoscopic |
| Bats [24] | 2008 | France | 43 | 0.70 | 0.37 | Blue dye | Cervical | H&E,IHC | Laparoscopic |
| Li [25] | 2009 | China | 31 | 0.74 | 0.45 | Blue dye | Uterine | H&E | Open |
| Mais [26] | 2010 | Italy | 34 | 0.62 | NR | Blue dye | Cervical | H&E,IHC | Laparoscopic/Open |
| Qu [27] | 2010 | China | 18 | 0.72 | NR | Blue dye | Uterine | H&E | Open |
| Ballester [28] | 2011 | France | 125 | 0.89 | 0.62 | Tc-99m+blue dye | Cervical | H&E,IHC | Laparoscopic |
| Holloway [29] | 2012 | United States | 35 | 0.97 | 0.77 | ICG /blue dye | Cervical | H&E,IHC | Robotic |
| Rossi [30] | 2012 | United States | 20 | 0.85 | NR | ICG | Cervical | H&E | Robotic |
| Solima [31] | 2012 | Italy | 80 | 0.95 | NR | Tc-99m+blue dye | Uterine | H&E,IHC | Open |
| Ballester [32] | 2013 | France | 103 | 0.86 | NR | Blue dye | Cervical | H&E,IHC | Laparoscopic |
| Mosgaard [33] | 2013 | Denmark | 32 | 0.72 | NR | Tc-99m+blue dye | Uterine | H&E,IHC | Open |
| Torné [34] | 2013 | Spain | 74 | 0.74 | 0.19 | Tc-99m+blue dye | Uterine | H&E,IHC | Laparoscopic |
| Vidal [35] | 2013 | France | 66 | 0.62 | 0.35 | Blue dye | Cervical | H&E,IHC | Laparoscopic |
| Desai [36] | 2014 | United States | 120 | 0.86 | 0.52 | Blue dye | Cervical | H&E,IHC | Robotic |
| Kadkhodayan [37] | 2014 | Iran | 24 | 0.88 | 0.67 | Tc-99m+blue dye | Cervical | H&E | NR |
| López-De [38] | 2014 | Spain | 50 | 0.92 | 0.34 | Tc-99m+blue dye | Cervical | H&E | Laparoscopic |
| Mucke [39] | 2014 | Germany | 31 | 0.90 | 0.52 | Tc-99m+blue dye | Cervical | H&E,IHC | Laparoscopic |
| Raimond [40] | 2014 | France | 156 | 0.87 | 0.57 | Tc-99m+blue dye | Cervical | H&E,IHC | Open |
| Allameh[41] | 2015 | Iran | 15 | 0.80 | NR | Blue dye | Uterine | H&E,IHC | Open |
| Eitan [42] | 2015 | Israel | 74 | 0.62 | 0.35 | Blue dye | Cervical | H&E | Robotic |
| Farghali [43] | 2015 | Egypt | 93 | 0.73 | NR | Blue dye | Uterine | H&E,IHC | Open |
| How [44] | 2015 | Canada | 100 | 0.92 | 0.76 | Tc-99m+blue dye/ICG/blue dye | Cervical | H&E,IHC | Robotic |
| Rajanbabu [45] | 2015 | India | 20 | 0.90 | 0.65 | ICG | Cervical | H&E | Robotic |
| Touhami [46] | 2015 | Canada | 19 | 0.79 | NR | Tc-99m+blue dye | Cervical | H&E,IHC | Laparoscopic |
| Buda [47] | 2016 | Italy | 118 | 0.90 | 0.58 | Tc-99m+blue dye/ICG/blue dye | Cervical | H&E,IHC | Laparoscopic |
| Ehrisman [48] | 2016 | United States | 36 | 0.83 | 0.56 | Blue dye | Cervical | H&E | Laparoscopic/Robotic |
| Elisei [49] | 2016 | Italy | 40 | 0.90 | 0.65 | Tc-99m+blue dye | Cervical | H&E,IHC | Laparoscopic |
| Markus [50] | 2016 | Switzerland | 24 | 0.75 | NR | Tc-99m+blue dye | Cervical | H&E,IHC | Laparoscopic |
| Martinelli [51] | 2016 | Italy | 57 | 0.89 | 0.67 | ICG | Uterine | H&E,IHC | Laparoscopic |
| Paley [52] | 2016 | United States | 123 | 0.97 | 0.80 | ICG | Cervical | H&E,IHC | Robotic |
| Papadia [53] | 2016 | United States | 75 | 0.96 | 0.88 | ICG | Cervical | H&E,IHC | Laparoscopic |
| Schiavone [54] | 2016 | United States | 48 | 0.83 | 0.71 | ICG | Cervical | H&E,IHC | Laparoscopic |

Abbreviations: DR, detection rate; H&E, hematoxylin and eosin; ICG, indocyanine green; IHC, immunohistochemistry; NR, not reported; SLN, sentinel lymph node; Tc-99m, technecium-99.
