# Supplementary material for: Sentinel lymph node mapping in endometrial cancer: a systematic review and meta-analysis
Source: Oncotarget. 2017 Mar 29;8(28):46601–10. doi: 10.18632/oncotarget.16662 (PMC5542296; doi:10.18632/oncotarget.16662)
Supplement: Supplementary file 3 [file oncotarget-08-46601-s003.doc]

**Appendix table 1: Quality assessment of included studies (The QUADAS-2 tool)**

|  | **Patient selection** | |  | **Index test** | |  | **Reference standard** | |  | **Flow and timing** | |  |
| --- | --- | --- | --- | --- | --- | --- | --- | --- | --- | --- | --- | --- |
| **Author, year** | **Was a**  **consecutive or random sample**  **of patients**  **enrolled?** | **Was a case-**  **control design**  **avoided? Did**  **the study avoid**  **inappropriate**  **exclusions?** |  | **Were the index**  **test results**  **interpreted**  **without**  **knowledge of**  **the results of**  **the reference**  **standard?** | **If a threshold**  **was used,**  **was it**  **prespecified?** |  | **Is the reference**  **standard likely**  **to correctly**  **classify the**  **target**  **condition?** | **Were the**  **reference**  **standard**  **results**  **interpreted**  **without**  **knowledge of**  **the results of**  **the index test?** |  | **Was there an**  **appropriate**  **interval**  **between the**  **index test and**  **reference**  **standard?** | **Did all patients**  **receive the**  **same reference**  **standard?** | **Were all**  **patients**  **included in**  **the analysis?** |
| Burke 1996 [11] | Y | Y |  | N | NR |  | Y | N |  | NR | Y | Y |
| Pelosi 2003 [12] | Y | Y |  | N | NR |  | Y | N |  | NR | Y | Y |
| Holub 2004 [13] | Y | Y |  | N | NR |  | Y | N |  | NR | Y | Y |
| Lelievre 2004 [14] | Y | Y |  | N | NR |  | Y | N |  | NR | Y | Y |
| Niikura 2004 [15] | Y | Y |  | N | NR |  | Y | N |  | NR | Y | Y |
| Basta 2005 [16] | NR | Y |  | N | NR |  | Y | N |  | NR | Y | Y |
| Gien 2005 [17] | Y | Y |  | N | NR |  | Y | N |  | NR | Y | Y |
| Maccauro 2005 [18] | Y | Y |  | N | NR |  | Y | N |  | NR | Y | Y |
| Altgassen 2007 [19] | Y | Y |  | N | NR |  | Y | N |  | NR | Y | Y |
| Delaloye 2007 [20] | Y | Y |  | N | NR |  | Y | N |  | NR | Y | Y |
| Lopes 2007 [21] | Y | Y |  | N | NR |  | N | N |  | NR | Y | Y |
| Yan 2007 [22] | Y | Y |  | N | NR |  | Y | N |  | NR | Y | Y |
| Ballester 2008 [23] | Y | Y |  | N | NR |  | Y | N |  | NR | Y | Y |
| Bats 2008 [24] | Y | Y |  | N | NR |  | Y | N |  | NR | Y | Y |
| Li 2009 [25] | NR | Y |  | N | NR |  | Y | N |  | NR | Y | Y |
| Mais 2010 [26] | Y | Y |  | N | NR |  | Y | N |  | NR | Y | Y |
| Qu 2010 [27] | Y | Y |  | N | NR |  | Y | N |  | NR | Y | Y |
| Ballester 2011 [28] | Y | Y |  | N | NR |  | Y | N |  | NR | Y | Y |
| Holloway 2012 [29] | Y | N |  | N | NR |  | Y | N |  | NR | Y | N |
| Rossi 2012 [30] | Y | Y |  | N | NR |  | Y | N |  | NR | N | Y |
| Solima 2012 [31] | Y | N |  | N | NR |  | Y | N |  | NR | Y | N |
| Ballester 2013 [32] | Y | Y |  | N | NR |  | Y | N |  | NR | Y | Y |
| Mosgaard 2013 [33] | Y | Y |  | N | NR |  | Y | N |  | NR | Y | Y |
| Torné 2013 [34] | Y | Y |  | N | NR |  | Y | N |  | NR | Y | Y |
| Vidal 2013 [35] | Y | Y |  | N | NR |  | Y | N |  | NR | Y | Y |
| Desai 2014 [36] | Y | Y |  | N | NR |  | N | N |  | NR | N | Y |
| Kadkhodayan 2014 [37] | Y | N |  | N | NR |  | Y | N |  | NR | Y | Y |
| Lo´pez-De 2014 [38] | Y | Y |  | N | NR |  | Y | N |  | NR | Y | Y |
| Mucke 2014 [39] | Y | Y |  | N | NR |  | Y | N |  | NR | Y | Y |
| Raimond 2014 [40] | NR | Y |  | N | NR |  | Y | N |  | NR | Y | N |
| Allameh 2015 [41] | Y | Y |  | N | NR |  | Y | N |  | NR | Y | Y |
| Eitan 2015 [42] | Y | Y |  | N | NR |  | N | N |  | NR | Y | Y |
| Farghali 2015 [43] | NR | Y |  | Y | NR |  | N | Y |  | NR | N | Y |
| How 2015 [44] | Y | N |  | N | NR |  | Y | N |  | NR | Y | Y |
| Rajanbabu 2015 [45] | Y | Y |  | N | NR |  | Y | N |  | NR | Y | Y |
| Touhami 2015 [46] | Y | Y |  | N | NR |  | Y | N |  | NR | Y | Y |
| Buda 2016 [47] | Y | Y |  | N | NR |  | Y | N |  | NR | Y | Y |
| Ehrisman 2016 [48] | NR | Y |  | N | NR |  | N | N |  | NR | Y | Y |
| Elisei 2016 [49] | NR | Y |  | N | NR |  | Y | N |  | NR | Y | Y |
| Markus 2016 [50] | NR | Y |  | N | NR |  | Y | N |  | NR | Y | Y |
| Martinelli 2016 [51] | Y | Y |  | N | NR |  | Y | N |  | NR | Y | Y |
| Paley 2016 [52] | Y | Y |  | N | NR |  | N | N |  | NR | Y | Y |
| Papadia 2016 [53] | Y | Y |  | N | NR |  | N | N |  | NR | Y | Y |
| Schiavone 2016 [54] | Y | N |  | N | NR |  | N | N |  | NR | N | Y |

Abbreviations: N, no; NR, not reported; Y, yes.
